# Virulent Bacteria as Inflammatory and Immune Co-Factor in Colon Carcinogenesis: Evidence From Two Monozygotic Patients and Validation in CRC Patient and Healthy Cohorts

**DOI:** 10.3389/fcimb.2021.749750

**Published:** 2021-11-04

**Authors:** Iradj Sobhani, Emma Bergsten, Cecile Charpy, Mathias Chamaillard, Denis Mestivier

**Affiliations:** ^1^EC2M3-EA7375, Research Team, Université Paris Est Creteil-UPEC, Paris and Creteil, France; ^2^Department of Gastroenterology, Henri Mondor Hospital, Assistance Publique Hopitaux de Paris (APHP), Paris and Creteil, France; ^3^Oncomix, Bacterial Toxins Unit Department of Microbiology- Institut Pasteur de Paris-France, Paris and Creteil, France; ^4^Department of Pathology Henri Mondor Hospital, Assistance Publique Hopitaux de Paris (APHP), Paris and Creteil, France; ^5^INSERM - Institut Pasteur Lille 59019, Lille, France; ^6^Bioinformatic Platform Institut de Recherche, Créteil, France

**Keywords:** cancer, colon, microbiota, immune cells, virulent bacteria

## Abstract

Colorectal carcinoma (CRC) is a common disease, the incidence of which is increasing according to Western lifestyle; it remains to have a poor prognosis. Western nutriments are presumed to induce mild inflammation within the colonic mucosa, resulting in the accumulation of DNA alterations in colonocytes through a multistage carcinogenesis process. This suggests that most CRCs are related to the environment. Of interest, fecal microbiota composition has been shown yielding a novel approach regarding how environment changes may impact health and disease. Here, we compare whole shotgun metagenomic gut microbiota of two monozygotic twin sisters, one of whom is suffering from an advance colorectal tumor with a profound disequilibrium of the composition of the gut microbiota due to the overexpression of virulent bacteria such as *E. coli, Shigella, and Clostridium* species in the colon cancer patient’s feces contrasting with low levels of bacterial species such as *Faecalibacterium* and *Akkermansia* usually enriched in the healthy adults’ microbial flora. The disequilibrium in microbiota of the CRC patient’s feces as compared to her monozygotic twin sister is linked to inflammatory and immune cell infiltrates in the patient’s colonic tissue. We speculate on the role of microbiota disequilibrium on the immune-tolerant cell infiltrate within CRCs.

## Introduction

Colorectal carcinoma (CRC) is one of the three most common cancers. There are more than 1.2 million new cases per year worldwide, accounting for about 600,000 deaths (Jemal et al., 2011). The disease is due to a multistep process involving gene- and environment-related factors (Vogelstein et al., 1988; Jass, 2007; Cancer Genome Atlas Network, 2012). Individuals with a first-degree relative affected by CRC have a two- to threefold higher risk of disease. Most CRCs are sporadic and less than 5% are hereditary (Foulkes, 2008), which suggests that environmental factors are the common underlying cause of CRC carcinogenesis. Studies in twins have enabled estimating putative roles of genetics and environment factors in the occurrence of CRC. Accumulation of DNA alterations (e.g., punctual DNA mutation, hypo or hypermethylation of anti-oncogene or oncogene promotors, respectively) corrupts key signaling pathways in the colonic mucosa in various carcinogenesis processes that involve hyperproliferation, the development of aberrant crypt foci (ACF), a transition from adenoma to carcinoma, tumor invasion, and metastasis (Vogelstein et al., 1988; Jass, 2007; Cancer Genome Atlas Network, 2012). The relative risk of colon cancer is higher in monozygotic than in dizygotic twins likely due to twofold numbers of genes shared in monozygotic relatives in addition to the environment they grow up in (Graff et al., 2017). Consequently, we analyzed gut microbiota as a boost for carcinogenesis in two monozygotic sisters and examined findings associated with tumor characteristics including inflammatory and immune patterns.

In the present individuals, one was considered as a case presenting with an invasive carcinoma, while her monozygotic twin sister, free of CRC, was considered as a control.

We showed that colonoscopy and pathology data were associated with both significantly different inflammatory or immune cell infiltrates within colonic mucosa and virulent bacteria species in feces. We further estimated which toxins are over- or underproduced in CRC patient’s stool according to the available metagenomic reads in stool.

## Cases

In April 2011, we examined Mrs. D., a 57-year-old woman without any personal or familial history of neoplasia, who had failed to participate in a mass screening program that was organized for asymptomatic individuals over the age of 50. She was referred to the gastroenterology department for a colonoscopy because of bowl disturbance and abdominal pain, anemia (10.9 dg/ml) with microcytosis, and a diminished ferritin level. She was not taking any medicine prior to these symptoms. Therefore, she was invited to join our prospective CCR1 cohort study (design and preliminary results published elsewhere) (Sobhani et al., 2011). The physical examination was unhelpful, and blood tests showed normal kidney function but altered hepatic liver tests. She underwent a colonoscopy, which was performed under general anesthesia. She provided informed consent to be enrolled in microbiota translational studies, including providing fresh stool samples 3 days before the colonoscopy. All additional investigations were performed on feces, the tumor, or normal tissues, which were conserved until analysis under FR-901 reference in our biology collection bank and dataset. An abdominal computed tomography (CT) scan revealed abnormal thickening of the sigmoid wall and more than five abnormal nodular tumors in the liver. After cleansing, the colonoscopy showed a 5-cm-diameter ulcerated tumor in the sigmoid that was suggestive of malignancy. A pathology tissue examination taken during colonoscopy demonstrated an invasive adenocarcinoma as assessed by H&S staining; this tumor was classified as Stage IV (according to TNM international classification) with *Kras* mutation and a low level of microsatellite instability (MSS). The patient underwent 5-fluorouracil, leucovorin, and oxaliplatin (Folfox4) chemotherapy plus anti VEGF-bevacizumab therapy. She told physicians that she had a monozygotic twin sister, Mrs. M., who was only taking hypertension drug therapy and had similarly failed to undergo screening for colon cancer. Thus, we asked Mrs. M., who had no digestive symptoms, to join our translational mass-screening trial (*Clinical Trial Vatnimad NCT01270360*). The patient similarly provided informed consent to allow research into her stool sample and deep analyses of her colonic tissues. Her colonoscopy showed two adenomatous polyps of 6 and 8 mm in the right flexure and left colonic sites, respectively, which were removed and conserved respectively in our biobank and dataset until analysis under FR-902P1 and FR-902P2 codes. Both twins were living with their parents until the age of 26, both were married, and neither had children. Mrs. D. died in September 2016 after fourth-line therapy failure, and Mrs. M.’s last examination in March 2017 showed no progressive disease in the intestine. Data from stool metagenomic analysis from these cases are compared to those of CRC patients and healthy controls as reported in our previous reported data (Sobhani et al., 2011; Zeller et al., 2014; Sobhani et al., 2019).

## Methods

### Patient’s Data File

All events during the follow-up period (at least 5 years from the baseline) were registered, and results of physical, biological, and imaging exams were recorded in our dataset.

### Microbiota Analysis

Stool samples were collected a few days before the day of colonoscopy and stored within 4 h for DNA extraction using the GNOME^®^ DNA Isolation Kit (MP Biomedicals, Santa Ana, CA) as previously described (Sobhani et al., 2011; Zeller et al., 2014). We submitted stool samples from the two individuals to whole-genome shotgun metagenomic sequencing. This was also performed on the whole cohort [see results in Refs (Zeller et al., 2014; Sobhani et al., 2019)]. We compared sequences with comprehensive reference databases for taxonomic assignment using consensual pipelines, as described elsewhere (Sobhani et al., 2019). To summarize, reads that went across quality checking (QC) and trimming were mapped to the NCBI non-redundant proteins database using diamond software (version 0.9.17) (Huson et al., 2016) and MEGAN6 (Buchfink et al., 2015), for taxonomy assignment and summarization at the species level. Less than 3% of each of these sequences were found to be unclassified or to correspond to contaminations by virus, Archaea, or Eukaryote. Briefly, about 1% to 2% of reads as assigned by whole metagenome sequencing were mapped with virulent bacteria protein levels, which could be estimated in two monozygotic women’s stool, and in our prospective survey cohort for CRC, they were estimated by using non-redundant protein database (VFDB) (Duvallet et al., 2017). Taxonomic assignment of these virulence factors was established by mapping patient’s reads from the CCR1 cohort (Zeller et al., 2014) including 47 patients with large (>1 cm) polyps and 57 CRC patients to the virulence factor database (VFDB) (Duvallet et al., 2017) using the bowtie2 mapping software. Mapped reads for each type of virulence factor were expressed in counts per million (CPM) for further comparisons. Then, raw data from twin sisters were compared with (mean and median) values from our prospective cohorts.

### Quantitative RT-PCR Analysis of Chemokines and Cytokines

Total mRNA was prepared from tumors (CRC or polyps) and homologous normal specimens using Trizol reagent and following the manufacturer’s protocol. Serial sections of 50-μm tissue were ground using stainless steel beads (5 mm) after adding cold Trizol. First-strand cDNA was synthesized in reverse transcriptase samples, each containing 2 μg of total RNA isolated from tissues 16 units/μl Moloney murine leukemia virus reverse transcriptase (Life Technologies), 4 μmol/L oligo(dT)12-18, and 0.8 mmol/L mixed dNTP (Amersham-Pharmacia Biotech). Quantitative PCR was performed in a LightCycler 2.0 System (Roche Diagnostics) using a SYBR Green PCR kit or a Hybridization Probe PCR kit from Roche Diagnostics. Appropriate sequences of the primers and probes were chosen as described (Aloulou et al., 2008; Le Gouvello et al., 2008). Glyceraldehyde-3-phosphate dehydrogenase (GAPDH), β2-microglobulin, or β-actin genes were considered housekeeping genes. The β2-microglobulin gene was used as the control HKG for normalizing the result because it displayed the most stable expression in both tumoral and nontumoral specimens (data not shown). All PCR conditions were adjusted to obtain equivalent optimal amplification efficiency in the different assays. Real-time PCR was used for relative quantification of IL1β, IL4, IL6, CXCR1, CD3, CD39, IL-8, IL-17, granzyme A and B, TNFα, TGFβ, perforin, RORγT, and FoxP3 mRNAs by using the SYBR Green PCR kit. Real-time PCR was used to provide absolute quantification of mRNAs by using a Hybridization Probe PCR kit. The copy number of the mRNA for all target genes and the HKG was determined by plotting the sample Ct values against the standard curve obtained with the corresponding “quantitative DNA standard” dilution series using LightCycler software 4.0. The abundance of the target gene mRNA was calculated as the copy number of the target gene per 106 copies of β2-microglobulin. All PCR experiments were done in duplicate. CD3, CD17, FoxP3, RORgt, and Pefroin were selected for characterization on IHC because they illustrate all lymphocyte infiltrate, THL17, helper, tolerant, and cytotoxic responses, respectively.

### Immunohistochemistry

Representative samples (*n* > 5 per tissue) from tumors (polyps and CRC) and normal homologous mucosa were selected for each case and paraffin-embedded 4-μm sections as described elsewhere (Aloulou et al., 2008; Le Gouvello et al., 2008). Briefly, for immunoreactive staining, the sections were pretreated (boiling in buffer, pH 6.1 or 8, 1:20 dilution of horse serum in PBS for 20 min; Vector Laboratories). The serum was then removed and incubated overnight with appropriate antibodies for 1 to 2 h depending on antibodies. The chromogen Sigma Fast 3,3′-diaminobenzidine (DAB; Sigma-Aldrich) was incubated with the tissue sections in the dark at room temperature for 4 min to visualize the antibody complex. The reaction was terminated by a water wash before being counterstained. For double staining IL17/CD3, the goat anti-human IL-17 antibody (diluted 1:40) was applied first and incubated for 2 h. Immunohistochemical staining was undertaken using a Vectastain AP kit according to the manufacturer’s instructions (Vector Laboratories, Burlingame, CA, USA) and visualization was done with Naphtol/Fast Red (Sigma-Aldrich). Subsequently, a rabbit anti-human CD3 (diluted 1:50 in PBS, Dako, France) was incubated for 1 h. Immunohistochemical staining was undertaken using ImmPRESS system (Vector Laboratories, Burlingame, CA, USA) and visualization was done with DAB substrate. Morphometry quantification of CD3, IL-17, FoxP3, and perforin, was performed according to QuPath program.

## Results

### Patients’ Outcome

Analysis of CCR1 cohort dataset revealed that the CRC patient case among two twin sisters died during the follow-up period while her monozygotic sister was alive and remained free of CRC.

### Microbiota Analysis

**Figure 1:**
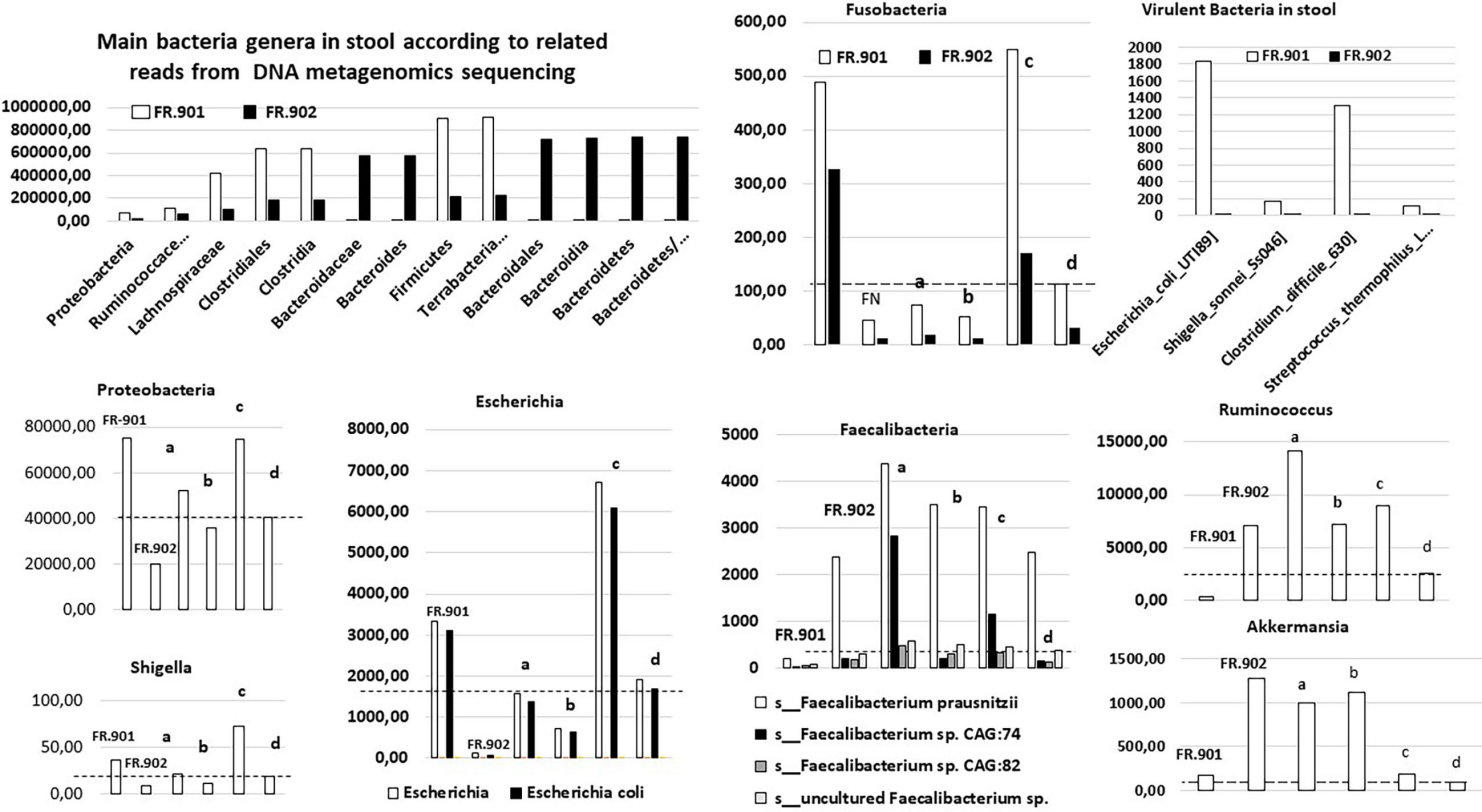
Levels of bacteria sequences, as assigned by whole metagenome sequencing [for methods see (Zeller et al., 2014; Sobhani et al., 2019)], by using the diamond software (MEGAN6) program and referring to the complete NCBI non-redundant protein database in two monozygotic twin sisters. The raw data are compared with values from our prospective cohorts, including patients with invasive CRC and those with precancerous polyps in the colon. The mean (a) and median values (b) in the adenomatous cohorts (*n* = 47), and the mean (c) and median values (d) in the CRC patient cohort (*n* = 187) are indicated for each bacteria; discontinued horizontal bars fit with the median value in the CRC patient cohort; FR-901 and FR-902 are the design code numbers of the CRC patient and her monozygotic sister, respectively.

Overall, bacteria sequences from stool did not show differences in total reads between CRC (*n* = 54,597,014) and no-CRC (*n* = 48,141,012) twin sisters when comparisons of taxa between the two sisters showed significant differences in terms of composition (**Figure 1**). Of note, principal component analysis (PCA) of all bacteria taxa between cohorts showed significant microbiota differences between CRC patients and colonic tumor-free individuals in the case and control investigated here.

With regard to the main bacteria genera usually associated with CRCs (for meta-analysis, see Ref. (Liu et al., 2019)), various species of *Bacteroides, Escherichia, Fusobacteria, Clostridia*, and *Proteobacteria* were enriched in the stool CRC compared with the control twin case. This mirrored a concomitant impoverishment of stool of CRC patients from various species of *Faecalibacterium* and *Ruminococcus* (**Figure 1**). By filtering all virulence factors from the gut bacterial microbiota (roughly 1% to 2% of all reads), we recorded 0.0004% in controls *versus* 0.002% in CRC patients, which represented 50-fold enrichment in CRC conditions. Quantitative analysis here unveiled the enrichment in virulence factors from *Shigella, Escherichia, Clostridia*, and *Streptococcus* species in the gut microbiota of patients from the CRC cohort, encompassing the twin suffering from CRC (**Figure 1**). This establishes that gut microbiota dysbiosis in colorectal cancer conditions leads to an enrichment of pathogenic bacteria encompassing *Shigella, Escherichia, Clostridia*, and *Streptococcus* species. This holds true from an example of monozygotic twin sisters up to the whole population.

### Cytokines and Chemokines in Tissues

**Figure 2:**
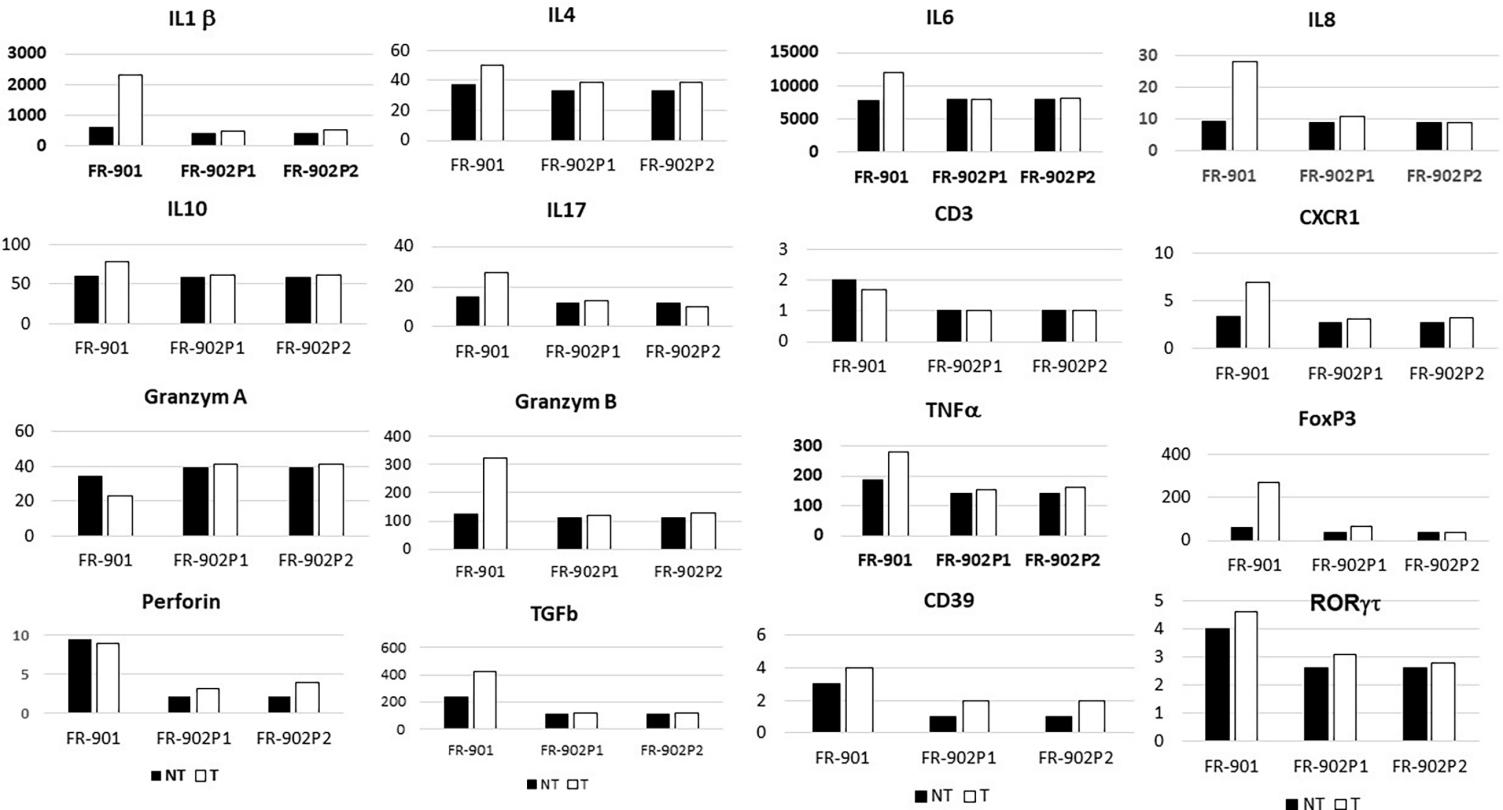
Levels of cytokines and chemokines’ transcripts, as assessed by qRT-PCR in normal and neoplastic (polyps and CRC) colonic tissues in two monozygotic sisters (in reference to housekeeping genes; for methods, see Le Gouvello et al., 2008). FR-901 and FR-902 are the design code numbers of the CRC patient and her monozygotic sister, respectively; P1 and P2 are design polyps in the no-CRC patient.

The mRNAs for IL1β, IL4, IL6, IL-8, CXCR1, IL10, TNFα, TGFβ, IL-17, FoxP3, RORγT, granzyme B, and perforin were significantly more abundant in the CRC samples than in homologous normal tissue while similar amounts of these cytokines were found in two adenomatous as compared to the normal homologous tissues (**Figure 2**). In tumor tissues, relationships were observed between perforin and granzyme B on the one hand and between IL-8 and CXCR1 on the other hand.

### Inflammatory and Immune-Cell Recruitment Within the Tumor Tissues From Twins

**Figure 3:**
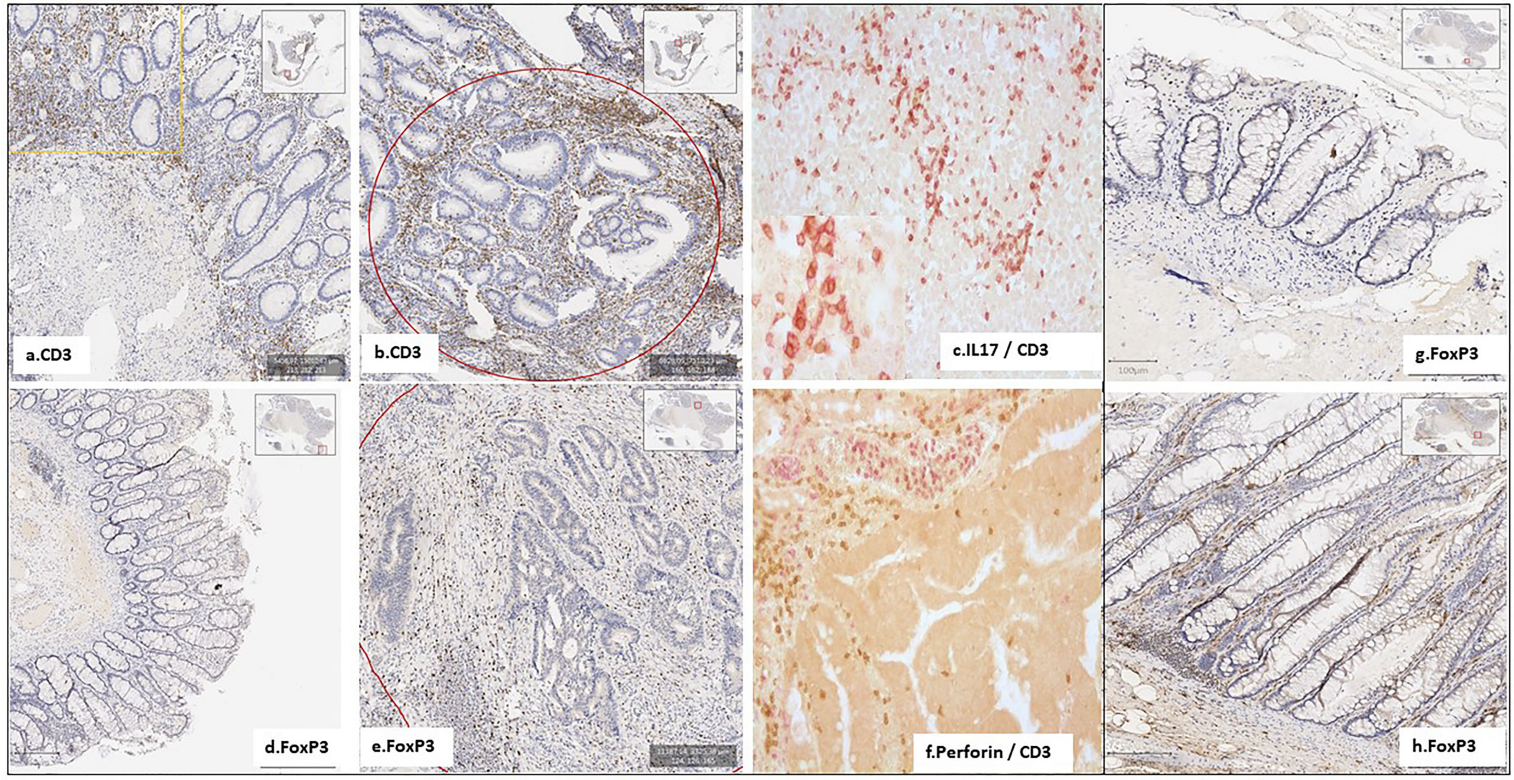
Few inflammatory and immune cells were detected in the homologous normal colonic mucosa, compared to the tumoral tissue, in the patient with CRC (FR.901) **(A–F)**. No active immune cells were detected in her monozygotic twin sister’s polyps (FR.902P20) **(G, H)**. Notably, the IL17 TH pathway is involved in CD4+ Foxp3+ IL-17+ cells, which were expanded in both CRC and adenomatous patients, with a trend towards elevated immunostained cells in CRC tissues.

Immunohistochemistry showed significantly higher CD3, CD3-IL17, FoxP3, and RORγT cells in colon cancer, but not in adenomatous-tissues as assessed by immune-stained cell counts. Furthermore, there is a trend toward augmentation of inflammatory cell infiltrates within the mucosa (**Figure 3**), with more mastocytes, monocytes, and polynuclear inflammatory cells, which were found infiltrating CRC tissues as compared to homologous normal tissues, while such differences regarding myeloid inflammatory cell infiltrates were not observed in two polyps of the no-CRC twin sister (results not shown). Despite these differences, markers of cytotoxic response, such as granzyme A and B, perforin, and RORγT as assessed by qRT-PCR quantifications, were not significantly enhanced in tumor tissues (**Figure 2**). Note that overall expressions of IL-17 and perforin immune reactive cells as assessed by immunohistochemistry were strongly correlated with those of cytokine release as assessed by qRT-PCR regardless whether they were measured in the normal or tumor tissues (*n* = 5 counts of each): *R* = 0.67, *p* = 0.01. Production of various molecules such as IL-6 and TNF-α in homologous normal tissues was associated with virulent bacteria abundances, suggesting strongly that mild inflammation in the normal tissue has a substantial macrophage involvement. We noticed more monocytes and polynuclear inflammatory cells infiltrating CRC tissues as compared to the homologous normal tissues. IL-17 and perforin immunoreactive cells were rarely detected in adenomatous or in normal tissues in the *lamina propria*. However, their numbers were always higher in colon cancer tissues than in homologous normal tissues.

## Discussion

Here, we report gut microbiota differences in two twin sisters, one of whom presenting with a metastatic colonic cancer and one with small benign polyps, a precancerous condition in the colon. The difference regarding fecal microbiota composition resembles those observed in CRC patients and controls in our CCR1 cohort. If risk factors were independent from the environment, these two monozygotic sisters would have developed the disease at the same age. The fact that the two sisters had different microbiota suggests that the environment might be involved in cancer development and should be considered as a potential risk contributor in the CRC occurrence. The interindividual differences that account for the environment’s role have been estimated to reach 15% (Graff et al., 2017). The impact of the environment may rely on the microbiota. This is the reason that we also indicated mean and median values in the prospective CCR1 cohorts including CRC patients and those with polyps. Indeed, based on PCA, these cohorts displayed different microbiota compositions in stool; bacteria compositions between the two twin sisters’ stool were similarly different. Furthermore, based on metagenomic gene proteins, sequences corresponding to the virulent toxins were found significantly different in two twin sisters. Despite findings such as small polyps in CRC-free out of two sisters, levels of such DNA sequences were close to that we observed in normal colonoscopy individuals through the CCR1 cohort when significant higher total virulent toxin protein gene sequences were found in CRC-affected sister’s stool.

Assuming that tissue-associated inflammation is a critical hallmark of cancer, and the resulting dysbiosis is a possible underlying cause of tissue-related inflammation, we screened both inflammatory and immune cells within tumors and homologous normal tissues. Indeed, *Helicobacter pylori* has been shown to be responsible for chronic inflammation in the gastric mucosa, which leads to fundic gland atrophy, hypochloridria, and gastric cancer (Uemura et al., 2001). In addition, innate and adaptive immune cells are features of cancer development and progression (Li et al., 2017), particularly making sporadic CRCs resistant to immune check point targets, which might be due to dysbiosis (Seow et al., 2016). Here, we show close associations between recruited inflammatory and immune cells with dysbiosis through toxin-producing bacteria.

Among total metagenomic DNA sequences, less than 1% of all reads were estimated to be designing virulent toxins; they constitute the main part of differences in two twin sisters as assessed by metagenomic gene reporter analysis; these individual values are close to those in all our cohorts with higher levels of virulent bacteria in the CRC patient than in the control. Since tissue-adherent bacteria are substantially influenced by those of the stool (Arthur and Jobin, 2013), we hypothesized that patients with overexpression of virulent bacteria might have higher inflammatory cell infiltrates in their tumoral mucosa. Thus, type and function of myeloid, macrophages, and T regulatory cells were analyzed in CRC tissues and in polyps by using IHC and qRT-PCR for the quantification of cells and chemokines, respectively. Production of molecules such as IL-6 and TNF-α, as linked with virulent bacteria abundances, strongly suggests that macrophages are mainly infiltrated in the tissues. Although we noticed more monocytes and polynuclear inflammatory cells infiltrating CRC tissues as compared to the homologous normal tissues, we could not determine whether M1 or M2 macrophages are involved in the CRC tissue. IL10 is an anti-inflammatory cytokine that acts through STAT3 for enhancing carcinogen-induced tumorigenesis in the intestine (Dedon and Tannenbaum, 2004). This cytokine is currently found at a higher level in CRC as compared to the CRC-free twin sister’s polyps. It has been shown that anti-inflammatory transcription factor Stat3 in macrophages enhances the risk of invasive adenocarcinoma (Dejea and Sears, 2016) in colon cancer. Briefly, changes in the macrophage-related cytokine levels in CRC tissue might cause tumor initiation by creating a mutagenic microenvironment through alterations in the microbiome and barrier functions (Dedon and Tannenbaum, 2004; Dejea and Sears, 2016). In addition, by screening immune cells, we clearly showed that TH1 and TH17 pathways were stimulated in CRC tissues, when patterns in the polyps resembled normal colonic tissue (**Figure 3**). Interestingly, microbiota considered as a source of stimulation of inflammatory response in the colonic mucosa revealed that less anti-inflammatory bacteria, such as *Faecalibacterium* and *Eubacterium*, were associated with overexpression of virulent bacteria such as *E. coli*, *Shigella*, and *Clostridium* species in the CRC patient’s (**Figure 1**) than in her CRC-free sister’s feces. Whether carcinogenesis is directly linked to this imbalance between pro- and anti-inflammatory bacteria or the result of a failed immune response to no microbial factors (e.g., chemical carcinogenic substances) has yet to be determined. As a fact referring to the precancerous colonic lesions, based on 16sRNA fecal prokaryote DNA sequencing in a series of more than 500 individuals undergoing colonoscopy, members of *Enterobacteriaceae* known to cause inflammation in the gastrointestinal tract have been shown to be overexpressed in conventional adenomas higher than 1 cm in diameter. However, the authors did not observe overabundance of these members in serrated polyps, which are precancerous lesions too (Peters et al., 2016). In another study, by using specific qPCR, Rezasoltani et al. (2018) show overabundance of some selected virulent bacteria such as *Porphyromonas* and enterotoxigenic *Bacteroides fragilis* with a diminution of probiotics such as *Bifidobacterum* in patients’ feces presenting with adenomatous tissues at colonoscopy. Tissue inflammatory cells have not been investigated in these studies. Whole metagenomic analysis conducted in our present study consistent with tissue inflammatory and immune cells analyses more strongly supports the hypothesis that virulent bacteria altogether may be involved in the colon carcinogenesis. Also, tissue-adherent bacteria should be identified to ensure those virulent bacteria are overabundant as compared to the normal or adenomatous tissues. Nevertheless, the core of virulent bacteria is linked to inflammation and immune response in the tumor likely through an epigenetic pathway (Sobhani et al., 2019). Indeed, several bacteria that are clustered within a community may be the cause of immunotolerant cell recruitment within tumor tissues. Overproduction of IL17 can lead to disease exacerbation. Relative concentrations of IL6 and TGFβ in tumor tissues balance the differentiation of coexisting CD4+ RORγt+ Th-17 cells and regulatory CD4+ FoxP3+ RORγt+ IL17-negative T cells. This ratio has been proposed to play a role in the regulation of immunoreactivity (Lochner et al., 2008). Additionally, T cells are components of the adaptive immune system. Thus, less immunogenic cancer cells escape T cells’ immune control and are able to survive a process called cancer immune editing (Teng et al., 2004; Aloulou et al., 2008), which consists of cancer cells adopting an immune-resistant phenotype (Seow et al., 2016). Then, cancer cells develop a mechanism able to prevent the local cytotoxic response of effector T cells as well as other cells, including NK cells and tumor-associated macrophages in the CRC twin case (as compared to her CRC-free sister’s polyps, FR-902P1 and P2). Tregs could be responsible for suppressing the priming activation and cytotoxicity of other effector immune cells, such as TH1, macrophages, and neutrophils despite their overexpression in the CRC tissue. This mechanism is orchestrated by the producer of immunosuppressive molecules, such as IL10 and TGFβ, currently overexpressed in CRC tissue. Tregs suppress T-cell activation, which has been shown to worsen patient outcomes (Aloulou et al., 2008). Increases in the mutation burden and heterogeneity of neoantigens *in vivo*, as well as the priming of a new anti-tumor T-cell repertoire, results from the inactivation of the DNA repair system in colorectal cancer (Dominguez-Valentin et al., 2020). Although stigmata of immune anti-tumor response (i.e., Perforin, CD39, RORγt) could be observed in the CRC patient’s tumor tissue in the present observation, cytotoxicity was not deemed likely to sufficiently imprint longer survival (Seow et al., 2016; Simoni et al., 2018).

Interestingly, more oral-cavity pathobionts have currently been detected in CRC patients’ microbiota, as reported in several other studies (Schmidt et al.). The species of *Fusobacterium*, such as *F. nucleatum*, which we found to be enriched in the CRC patient, has been shown to be involved in a tumor-immune evasion mechanism, through which tumors exploit the Fap2 protein of the bacterium to inhibit immune-cell activity (Gur et al., 2015). Together, the community of oral-cavity bacteria stimulates IL17-producing cells during periodontitis (Schmidt et al.). The main difference between our two patients’ microbiota concerned the virulent bacteria community (**Figure 1**). It is interesting to note that, among the virulent bacteria, T-helper 1 inflammatory responses in the gut (Gharaibeh and Jobin, 2019) in our CRC patient can be attributed to a bacterium such as *Klebsiella*, rather than to others such as *E. coli* or *C. difficile* that do not trigger a TH1 response. The diminished levels of *Rhuminococcus*, *Faecalibacteria*, and *Akkermansia* in the CRC patient’s stool, compared to her CRC-free sister, suggest that diminution of these bacteria may account for a favored immune-response shift from immunotoxicity to tolerance. Notably, *Bifidobacterium* bacterium known to protect against colonic neoplasia showed abundances similar to the two twin sisters’ stool; this suggests that this probiotic effect alone might not protect against transformation of adenomatous tissues into invasive carcinoma, a hypothesis consistent with cytokines of TH1, TH2, and TH17, which were detected in similar levels to those of the normal colonic mucosa of the two individuals (**Figure 2**). As a perspective for deeper investigations involving host and microbiota plasticity, one would suggest screening virulent-associated metabolites in feces and/or urines and characterize functions of tumor-infiltrating lymphocytes (O'Keefe, 2016; Xiao et al., 2020), which have not been studied here. In the future, a particular attention should be paid to the epigenetic pathways in host colonocytes and immune cells (Sobhani et al., 2019; Yates et al., 2021).

In conclusion, thanks to two monozygotic twin sisters’ data and biological analysis, we show that occurrence of colonic cancer is strongly related to the abundance of bacteria genes coding for pro-inflammatory toxin proteins in stool. This pattern is validated in two large cohorts with CRC and without CRC. We further report higher immune-tolerant cells within colonic tumor likely as a biological response to overproduction of virulent toxins. A metabolomic approach for measuring toxin proteins is necessary to validate this observation.

## Data Availability Statement

The original contributions presented in the study are included in the article/**Supplementary Material**. Further inquiries can be directed to the corresponding author.

## Ethics Statement

The studies involving human participants were reviewed and approved by CCR1 study and Vatnimad study Ile de France research ethical committee. The patients/participants provided their written informed consent to participate in this study. For more details see ClinicalTrials.gov Identifier: NCT01270360.

## Author Contributions

IS: design, collecting data, and manuscript preparation. EB: collecting data. CC: collecting data. DM: bioinformatics and statistical analyses MC: coordination in financial support and scientific advises. All authors contributed to the article and approved the submitted version.

## Funding

This study is performed with the financial support of Université Paris Est Creteil (UPEC), PHRC (Vatnimad) of French government as well as by SNFGE (French society of Gastroenterology Commad Support 2019) and the financial support from ITMO Cancer AVIESAN (Alliance Nationale pour les Sciences de la Vie et de la Santé, National Alliance for Life Sciences & Health) within the framework of the Cancer Plan (HTE201601) with the coordination of MC which was supported by INCA and executed through Institut Pasteur de Paris, APHP and UPEC.

## Conflict of Interest

The authors declare that the research was conducted in the absence of any commercial or financial relationships that could be construed as a potential conflict of interest.

## Publisher’s Note

All claims expressed in this article are solely those of the authors and do not necessarily represent those of their affiliated organizations, or those of the publisher, the editors and the reviewers. Any product that may be evaluated in this article, or claim that may be made by its manufacturer, is not guaranteed or endorsed by the publisher.
